# Dietary intake, intestinal infection, and safe drinking water among children with anemia in Peru: a cross-sectional analysis

**DOI:** 10.1186/s40795-021-00417-3

**Published:** 2021-06-03

**Authors:** Christopher M. Westgard, Luis A. Orrego-Ferreyros, Liz Franco Calderón, Alexandra M. Rogers

**Affiliations:** 1 Department of Research and Innovation, Elementos, Lima, Peru; 2 Department of Maternal and Child Health, Gillings School of Global Public Health, University of North Carolina at Chapel Hill, Chapel Hill, NC USA; 3 Faculty of Public Health and Administration, Universidad Peruana Cayetano Heredia, Lima, Peru; 4 Department of International Health, Bloomberg School of Public Health, Johns Hopkins University, Baltimore, MD USA

**Keywords:** Anemia, Nutrition, Diet, Iron, Intestinal infectious disease, Parasites, Water, Peru

## Abstract

**Background:**

Anemia is a major public health concern that is present in 41.7% of children under 5 worldwide. The prevalence of anemia in Peru was 43.6% in 2017, a decrease by only 6.8% in 8 years. Despite great efforts made by the government to reduce anemia by distributing free multi-micronutrient supplements and promote the consumption of iron rich foods, progress has been slow. The current study sought to better understand why the prevalence remains high by analyzing the dietary intake, incidence of intestinal infectious disease, and access to safe drinking water by children with anemia in Peru.

**Methods:**

A cross-sectional analysis was conducted using data from two national surveys that were combined by child ID. Descriptive statistics was analyzed to understand the experience of children with anemia in comparison to child without anemia. Logistic multivariate regression analyses were conducted to test the associations between anemia and dietary intake, intestinal infection, and access to safe drinking water.

**Results:**

The sample included 586 children between 6 and 35 months. The prevalence of anemia in this population was 53%. The portion of children that consumed sufficient iron to meet the recommendation for their age was 62%. Of the children with anemia, 52% consumed sufficient iron to meet their recommendation, vs. 72% of children without anemia (*p* < 0.001). The children with anemia were more likely to have an intestinal infection during the previous year (35% vs. 26%, *p* = 0.057) and less likely to have access to safe drinking water (77% vs. 86%, *p* = 0.002) than those without anemia. The logistic analysis revealed that having an intestinal infection increased the odds of having anemia (OR = 1.64, CI 95% [1.041–2.584]), and having access to safe drinking waters decreased the odds of having anemia (OR = 0.578, [0.334–0.998]).

**Conclusions:**

More than half of the children with anemia in Peru already consume sufficient iron to meet their daily requirement. However, they continue to have anemia, likely due to intestinal infection, such as diarrhea and parasites, from a lack of access to safe drinking water and hygienic practices.

## Background

Proper nutrition in children, especially during the first 1000 days, is critical for healthy growth and development [1–4]. However, in 2016, 41.7% of children under 5 globally suffered from anemia, commonly caused by micronutrient deficiencies [5]. The consequence of anemia on the health and economic development of communities are significant [6] Anemia can create significant developmental delays in children, weaken their immune system, and decrease the amount of energy they have available to grow and learn [7–9] A deeper understanding of anemia’s etiology in diverse contexts is needed to appropriately combat the disease and reduce its effects on children’s health and development.

Anemia is a multicausal problem created by micronutrient deficiencies, intestinal infections from parasites and bacteria, malaria, and genetic hemoglobin disorders [6]. Iron deficiency is the most common cause of anemia which accounts for almost half of all cases in children under 5 years of age worldwide [6]. Iron is an essential nutrient for hemoglobin and the production of red blood cells. Iron deficiency occurs when iron absorption into the blood does not meet the body’s iron requirements, due to poor diet or impaired absorption, decreasing the amount of hemoglobin in the blood below the threshold of healthy levels determined by age, sex, and other factors [7, 10]. The body’s need for iron is especially high during infancy and childhood, when the body experiences rapid growth.

The risk of iron deficiency anemia globally has been linked to various socioeconomic determinants, such as poor access to proper sanitation, unhygienic practices in the household, parents’ education level, family income, and family size [11–13]. Research in Peru has shown that childhood anemia is associated with poor intake of iron-rich foods, high prevalence of infectious diseases, a lack of safe water, low birth weight, and insufficient breastfeeding [14].

In Peru, 43.6% of children below 3 years old had anemia in 2017, a 6.8% reduction since 2009 [15]. Reducing childhood anemia has become a high priority of the Peruvian National Government. The Government has created a multisectoral plan that aims to reduce anemia in children under 3 by over 24% [16]. National programs have focused primarily on providing micronutrient supplements and educational campaigns to increase children’s intake of iron rich foods [17]. However, due to the multicausal nature of the disease, a multisectoral approach that goes beyond micronutrient supplementation and promoting dietary changes is needed.

The slow rate of decline of anemia in Peru in the face of extensive government efforts to reduce the prevalence merits a deeper look at the children with anemia to better understand their experience. The current prevention campaigns seem to suggest that increased consumption of iron will solve the problem of childhood anemia. The current study investigates this proposition by describing the dietary intake of children with anemia and analyzing the primary drivers of anemia independent of dietary intake. The study provides a greater understanding of the nutritional, health, and behavioral characteristics of children with anemia in Peru, to illuminate their needs and opportunities to create significant reduction in anemia.

## Methods

A cross-sectional analysis was conducted with data from two public databases combined by child ID. One of the databases included information from the national survey, *The Food and Nutrition Surveillance Survey for Stages of Life (FNSS), 2015* (In Spanish: Encuesta de Vigilancia Alimentaria y Nutricional por Etapas de Vida, VIANEV) [18]. The other database included administrative data from public health centers, called the*Health Benefits Report from the Integrated Health Services Systems of Comprehensive Health Insurance (ISHS), Ministry of Health 2015* (In Spanish: Reporte de Prestaciones de Salud del Sistema Integrado de Aseguramiento en Salud del Seguro Integral de Salud del Ministerio de Salud, SIASIS) [19]. The data extracted from the ISHS corresponded with the timeframe of the FNSS (2015) to ensure the indicators from both datasets were recorded during the same timeframe.

### The food and nutrition surveillance survey for stages of life (FNSS)

The FNSS is a nationally representative survey conducted by the National Center for Food and Nutrition, of the National Institute of Health (in Spanish: Centro Nacional de Alimentación y Nutrición). The survey utilized a cluster sampling survey design with randomized selection to represent the national population. The survey examined nutritional outcomes and dietary intake in children under 3 years.

Dietary intake was measured with a 24-h dietary recall on two non-consecutive days. The weight of each food consumed was estimated by weighting an approximately equivalent portion of the food with the survey participant. The dietary information was converted into the amount of nutrients consumed by the child during each day. To estimate the distribution of intake of nutrients, the survey used the software, *PC-SIDE*, developed by Iowa State University [20]. The intake of nutrients was compared to the dietary recommendations to meet estimated energy requirements (EER) by age and sex, defined by the Department of Nutrition for Health and Development of the World Health Organization [21–24]. The calculation determined if each participant met the nutrient requirements for their age of each nutrient category. The complete methodology is described in the survey’s final report [18].

The FNSS survey used a portable spectrophotometer to estimate hemoglobin concentration of the participants. The hemoglobin concentrations were used to diagnosis anemia using cut-off points defined by the World Health Organization. For children aged 6–59 months, the survey used the cut-off point defined by the World Health Organization of less than 11 g per liter [25]. Due to the method of measurement, it is not possible to distinguish between the type or cause of anemia. Iron-deficiency anemia is the most common in the population [26].

The FNSS provided information regarding the water source of households surveyed. The water source was defined as safe drinking water if it same from public water source such as public water piped into the household, shared water pipe outside of the home, or a community well [18]. The information is represented in the current study by the categorical variable, *access to safe drinking water*, in which 0 = no access to a safe drinking water source and 1 = the household has access to a safe drinking water source.

The FNSS provided information that is used by the current study to control for the effect of poverty of the household. The variable is not a key predictor of interest, but it is included in the logistic regression analysis to control for its influence. The variable, *basic needs met*, indicators if the home meets the basic needs of the family. The home does not meet the basic needs if it is constructed with non-structurally sound material (plastic, carboard, etc.), dirt floor, overcrowded, no toilet (indoor or outdoor), a child aged 6–12 does not attend school, or if the head of the household did not complete primary school.

The FNSS survey database provided the following variables; child met iron recommendations, child met micronutrient recommendations (iron, zinc, vitamin A), child met micronutrient and energy requirements (iron, zinc, vitamin A, and calories), anemia diagnosis, access to safe drinking water, basic needs met, sex, age, and area of residence (metropolitan Lima, urban or rural).

### The integrated health Services Systems of Comprehensive Health Insurance (ISHS)

The ISHS is a public database that contains administrative data reported by all public medical centers that accepts the public health insurance [19]. It provides information on the medical care provided to the population The database is available by request from the Ministry of Health of Peru. The illnesses reported in the databased, that were diagnosed and treated in the health centers, were categorized according to the International Classification of Diseases: Preparation of Short Lists for Data Tabulation [27]. The information of interest for this study from the ISHS database is the cases of intestinal infection or parasitic disease in child in 2016. The information is represented as a binary variable that indicates if the child was diagnosed with an intestinal infectious disease (1 = at least 1 reported infection) or if the child has had no diagnosis of an intestinal infectious disease at a public health center during the year 2015–2016 (0 = no reported infection). The category of “intestinal infectious disease’ for this study includes bacterial intestinal infections, viral intestinal infections, and parasitic intestinal infections.

The FNSS and ISHS survey databases were combined by the Department of Information Technology of the Integrated Health Insurance Program, upon request by the authors. The public institution combined the FNSS and ISHS survey databases with the participants’ national ID number. The institution maintains confidentiality of the information and does not share any identifiable information of the participants.

### Analysis

Descriptive statistics were analyzed to better understand the experience of children with anemia in comparison to child without anemia. The differences between the two groups were compared with a Chi-square test to identify if the differences were statistically significant. Two logistic analyses were conducted to assess the strength of association between the key predictors and anemia in children in Peru. The first model assessed the association between anemia and intestinal infections, intake of iron, a poverty measure, and sex. The second model assessed the association between anemia and access to safe drinking water, intake of iron, a poverty measure, and sex. The model with intestinal intake and model with access to safe drinking water are analyzed separately because intestinal infection is a mediator between safe drinking water and anemia, and thus blocks the flow of association through the causal path [28].

Any cases that had omitted variables were not included in the analysis. The analysis adjusted for sampling design and clustering. The analysis was conducted with STATA/SE 16.1 [29].

## Results

The FNSS included 586 participants between 6 to 35 months with no omitted variables of interest of the current study. The sample was 53% male with an average age of 20 months. The portion living in Metropolitan Lima was 35, 27% in Urban areas, and 38% in Rural areas. The basic needs of the household were not met in 36% of the families (defined by measure of poverty in Peru) [30]. The population had a 53% prevalence of childhood anemia, with the highest prevalence found in rural areas (Rural: 64%, Urban: 55%, Metropolitan Lima: 40%). The portion of respondents that had access to a safe water source was 82%. These findings, and the portion of children that met their recommendation for iron intake, micronutrient intake, and micronutrient and energy intake is displayed in Table 1.

**Table 1:** Characteristics of study population

	N (%)
**Characteristics of Study Population**	
Sex^b^	
Male	299 (52.6)
Female	269 (47.4)
Age (months)^b^	20 ± 8.5^a^
Area of residence^b^	
Metropolitan Lima	201 (35.4)
Urban	154 (27.1)
Rural	213 (37.5)
Basic needs met^b^	
No	211 (36.1)
Yes	375 (63.9)
Childhood Anemia^b^	
No	274 (46.8)
Yes	312 (53.2)
**Dietary uptake**	
Children that meet recommendation for iron^b^	
No	218 (38.4)
Yes	350 (61.6)
Children that meet recommendation for iron, zinc and vitamin A^b^	
No	282 (48.1)
Yes	304 (51.9)
Children that meet recommendation for iron, zinc, vitamin A, protein and, energy^b^	
No	342 (58.4)
Yes	244 (41.6)
Access to safe drinking water^b^	
No	106 (18.1)
Yes	480 (81.9)

^a^Mean ± standard deviation

^b^Reported from FNSS

The ISHS survey included 388 children aged 6–36 months that were also included in the FNSS survey. The prevalence of children who had an intestinal infectious disease in the last year was 31%. The comparison between children with anemia and those without anemia revealed significant differences in nutrient intake, incidence of intestinal infection, and access to safe drinking water. Among the children with anemia, 52% consume the recommended amount of iron for their age, while of those without anemia 72% consume the recommended amount of iron. The comparison can be seen in Table 2.

**Table 2:** Group comparison between those with anemia and those without anemia

Characteristic	Children with anemia		*p*-value
	No	Yes	
	n (%)	n (%)	
Area of residence			< 0.001
Metropolitan Lima	120 (59.7)	81 (40.3)	
Urban	70 (45.5)	84 (54.5)	
Rural	76 (35.7)	137 (64.3)	
Children that meet recommendation for iron			< 0.001
No	74 (27.8)	144 (47.7)	
Yes	192 (72.2)	158 (52.3)	
Children that meet recommendation for iron, zinc, and vitamin A			< 0.001
No	107 (39.1)	175 (56.1)	
Yes	167 (60.9)	137 (43.9)	
Children that meet recommendation for iron, zinc, vitamin A, protein and energy			0.001
No	140 (51.1)	202 (64.7)	
Yes	134 (48.9)	110 (35.3)	
Children with an intestinal infectious disease			0.057
None	121 (73.8)	143 (64.7)	
At least 1	43 (26.2)	78 (35.3)	
Access to safe drinking water			0.002
No	39 (14.1)	73 (23.4)	
Yes	235 (85.9)	239 (76.6)	

The analysis also revealed that of the children who did not have access to safe drinking water, 47.4% had an intestinal infection, while 27% of those with safe drinking water had an intestinal disease. To treat water in the household, 85.8% (332) of the participants who do not have access to safe drinking water report boiling their water to treat it, 3.9% (15) report treating the water with chlorine, and 0.8% (3) report treating another way.

The first logistic multivariant regression analysis revealed that having an intestinal infection increases the odds of having anemia (OR = 1.64, CI 95% [1.041–2.58], *p* = 0.033). The second logistic multivariant regression analysis revealed that having access to clean drinking water decreases the odds of anemia (OR = 0.578, CI 95% [0.334–0.998], *p* = 0.049). The results of the logistic regressions are displayed in Table 3.

**Table 3:** Logistic Regression Analysis on anemia

*N* = 398	Odds Ratio	Standard Error	*P*-value	95% Confidence Interval
**Analysis 1:**				
Iron Requirements	0.505	0.109	0.002	0.331–0.770
Intestinal Infectious Disease	1.640	0.380	0.033	1.041–2.584
Male	1.182	0.250	0.428	0.782–1.789
Poverty	1.473	0.323	0.077	0.959–2.262
Intercept	1.378	0.310	0.155	0.886–2.143
**Analysis 2:**				
Iron Requirements	0.539	0.116	0.004	0.353–0.822
Safe Drinking Water	0.578	0.161	0.049	0.334–0.998
Male	1.195	0.252	0.399	0.789–1.801
Poverty	1.364	0.298	0.155	0.889–2.093
Intercept	2.469	0.771	0.004	1.339–4.551

## Discussion

The results display that 52.3% of children with anemia in Peru already consume the recommended amount of iron in their diet. Similar results were found in a study in Peru in 2014, which showed that 51% of children under 3 met their iron uptake requirements and 60% met their energy intake requirements [31]. In the study, they did not assess how the diet related to the prevalence of anemia. It could have been assumed that the children with anemia were those that did not consume the recommended levels of micronutrients, and so promotion of multi-micronutrient supplements and increased consumption of iron rich foods could solve the problem of anemia.

The causes of anemia include poor nutrient intake, infectious diseases, parasites, inflammation, and hemoglobinopathies [6]. The risk of anemia is greater when a population has frequent infection and does not have safe drinking water or practice good hygiene, and does not have access to education and economic opportunities [19]. We see in the current study that nearly half of the children with anemia consume the recommended amount of nutrients, but it is not being appropriately absorbed and converted into hemoglobin. We must look beyond diet to significantly reduce the drivers of anemia. By integrating medical care data with the national nutrition survey, we were able to investigate other drivers of anemia beyond nutrition uptake. Intestinal infectious diseases, including diarrhea and helminth infections, have been linked to the development of anemia [32–36]. The logistic regression displayed that the presence of an intestinal infection increases the odds of having anemia (OR = 1.64, *p* = 0.033), with meeting iron requirements included in the model (OR = 0.51, *p* = 0.002). The results help explain why so many children have anemia, following years of national campaigns to provide micronutrient supplements and education campaigns to increase iron uptake. Strategies that do not address the presence of disease in children will continue to produce limited effects on the reduction of anemia [24–26]. We must look at ways to prevent diarrhea, infectious diseases, and helminth infections in order to reduce children’s risk of anemia.

Access to safe drinking water is associated with a lower risk of diarrhea and helminth infections; and diarrhea and helminth infections can cause anemia [32, 33, 37]. The current study found that children with access to safe drinking water are less likely to have anemia (OR = 0.58, *p* = 0.049). However, in a country that is prioritizing the reduction of anemia, it is worrisome that 29% of the rural population still do not have access to safe drinking water (2019) [15]. This study highlights the importance of improving access to safe drinking water and promoting proper sanitation and hygiene to reduce children’s risk for anemia.

The study has limitations created by the nature of the data included in the analysis. Because the data are cross-sectional, we cannot establish causality nor rule out the possibility of reverse causality. The children could have experienced a change in their diet following a diagnosis of anemia by the health center or self-diagnosis by their caregivers. However, given that anemia evaluations are infrequent in Peru and extremely difficult to diagnosis by observing the condition of the child, it is unlikely the diet at the time of the survey was influenced by a previous diagnosis of anemia. Additionally, the fact remains that if the children were living in sanitary conditions the change in diet should have pulled them out of anemia (assuming the diet had been maintained for weeks prior to the survey). Another limitation of the data is the measurement of intestinal infectious disease because it includes only the cases that are reported in the health center, and thus misses many incidences of diseases that are not reported due to poor access to health services and poor reporting in the health center [38]. The number of children that had experienced an intestinal infectious diseases is expected to be much higher. Nutritional surveys that utilize self-reporting also have their limitations that must be considered, included over or under reporting the amount of food consumed during the previous day. However, the FNSS attempted to reduce the bias by surveying each home on two separate, non-consecutive days. Finally, the FNSS did not include information on breastfeeding, which is a significant limitation when calculating the intake of nutrients by children less than 2 years of age.

## Conclusion

The present study has provided us with the opportunity to explore more deeply the dietary and disease profile of children with anemia in Peru. The results give us a better understanding of the factors that are helping to maintain a high prevalence of anemia in children below 3 years of age. The analysis hopes to show that the causes of anemia are diverse, and thus demand a diverse national strategy. The problem cannot be solved while children continue to live with poor sanitation and high incidence of disease. The promotion of iron consumption and micronutrient supplements alone will not be enough.

Our study has found that access to safe drinking water and incidence of intestinal infection are significantly associated with anemia. To reduce the incidence of intestinal infections and anemia, national policies to improve access to safe drinking water and sanitation must be implemented. It is essential that any approach to combat anemia in Peru focuses not only on the dietary causes of anemia, but also addresses the social determinants that affect children’s health.

## Data Availability

The deidentified dataset supporting the conclusions of this article is available in the figshare repository, 10.6084/m9.figshare.12574001. Public access to the database is open.

## Abbreviations

FNSS: Food and Nutrition Surveillance Survey for Stages of Life
ISHS: Health Benefits Report from the Integrated Health Services Systems of Comprehensive Health Insurance
SIASIS: Reporte de Prestaciones de Salud del Sistema Integrado de Aseguramiento en Salud del Seguro Integral de Salud del Ministerio de Salud
VIANEV: Encuesta de Vigilancia Alimentaria y Nutricional por Etapas de Vida
